# An Improved Refined Composite Multivariate Multiscale Fuzzy Entropy Method for MI-EEG Feature Extraction

**DOI:** 10.1155/2019/7529572

**Published:** 2019-03-28

**Authors:** Mingai Li, Ruotu Wang, Jinfu Yang, Lijuan Duan

**Affiliations:** ^1^Faculty of Information Technology, Beijing University of Technology, Beijing 100124, China; ^2^Beijing Key Laboratory of Computational Intelligence and Intelligent System, Beijing University of Technology, Beijing 100124, China

## Abstract

Feature extraction of motor imagery electroencephalogram (MI-EEG) has shown good application prospects in the field of medical health. Also, multivariate entropy-based feature extraction methods have been gradually applied to analyze complex multichannel biomedical signals, such as EEG and electromyography. Compared with traditional multivariate entropies, refined composite multivariate multiscale fuzzy entropy (RCmvMFE) overcomes the defect of unstable entropy values caused by the scale factor increase and is beneficial towards obtaining richer feature information. However, the coarse-grained process of RCmvMFE is mean filtered, which weakens Gaussian noise and is powerless against random impulse noise interference. This yields poor quality feature information and low accuracy classification. In this paper, RCmvMFE is improved (IRCmvMFE) by using composite filters in the coarse-grained procedure to enhance filter performance. Median filters are employed to remove the impulse noise interference from multichannel MI-EEG signals, and these filtered MI-EEGs are further smoothed by the mean filters. The multiscale IRCmvMFEs are calculated for all channels of composite filtered MI-EEGs, forming a feature vector, and a support vector machine is used for pattern classification. Based on two public datasets with different motor imagery tasks, the recognition results of 10 × 10-fold cross-validation achieved 99.43% and 99.86%, respectively, and the statistical analysis of experimental results was completed, showing the effectiveness of IRCmvMFE, as well. The proposed IRCmvMFE-based feature extraction method is superior compared to entropy-based and traditional methods.

## 1. Introduction

Brain-computer interface (BCI) is a new type of human-computer interaction technology that enables the brain to control external devices [1, 2]. Motor imagery electroencephalogram- (MI-EEG-) based BCI has great prospects in the field of rehabilitation medical engineering. One of the key technologies of BCI is the ability to effectively extract features from complex multichannel MI-EEG signals.

Previous studies focus on time-frequency methods in MI-EEG feature extraction, including wavelet transform (WT) [3], discrete WT (DWT) [4], Hilbert–Huang transform (HHT) [3], dual-tree complex WT (DTCWT) [5], empirical mode decomposition (EMD) [6], and common spatial pattern- (CSP-) based methods, including CSP, filter bank CSP (FBCSP), discriminant FBCSP (DFBCSP), sparse FBCSP (SFBCSP), and spectrally weighted CSP (SWCSP) [7–9]. With the development of nonlinear dynamics, entropy-based methods have been widely utilized in the analysis of biomedical signals. Specifically, the most prevalent methods are approximate entropy (ApEn) and sample entropy (SampEn) because of the power these methods have to quantify the complexity of a time series [10–12]. Nevertheless, sensitivity to selected parameters will lead to entropy mutation. To solve this weakness, fuzzy entropy (FE) was proposed for EEG analysis, where fuzzy membership functions replace Heaviside functions [13–15]. Research shows that FE alleviates the problem of entropy mutation; however, these methods analyze at a single scale, which loses useful information. Therefore, multiscale SampEn (MSE) [16–19], multiscale FE (MFE) [20, 21], and improved MFE (IMFE) [22] were put forward to explore deeper information. Unfortunately, using larger scale factors for short time series may cause inaccurate MSEs and MFEs. To achieve more reliable results, composite MFE (CMFE) [23, 24] was developed as a rolling bearing fault detection method.

Although the above univariate methods have shown good performance, they are only suitable for single-channel recording analyses. They fail to measure multichannel data synchronously and ignore the dynamic characteristics across channels [25]. So, SampEn was extended to produce multivariate SampEn (mvSE) [26] and multivariate MSE (mvMSE) [26–29] to analyze multichannel signals more effectively. Considering the disadvantages of SampEn in mvSE and mvMSE, multivariate FE (mvFE) and multivariate MFE (mvMFE) [30, 31] were yielded by replacing SampEn with FE. Recently, as an improvement of mvMFE, a refined composite mvMFE (RCmvMFE) was proposed to analyze fault signals and biomedical signals [32, 33]. In RCmvMFE, the entropy stability is improved and the signals' length sensitivity is reduced. However, the coarse-grained process of RCmvMFE is a mean filter that smoothens signals but does not eliminate random impulse noise interference. It is inevitable to produce high-amplitude electrooculogram and electromyography interference during the acquisition of MI-EEG. This is not conducive to extracting valid feature information from multichannel MI-EEG signals. In this paper, improved RCmvMFE (IRCmvMFE) is developed by combining median [34] and mean filters in the coarse-grained process to further improve filter effect, i.e., first the median filter is applied to each channel to remove pulse interference, and then the mean filter is used for further smoothing. Subsequently, IRCmvMFE is proposed to extract features from multichannel MI-EEG signals. The experimental research shows the effectiveness of IRCmvMFE.

The rest of the paper is described as follows: Section 2 introduces the process of extracting MI-EEG features using IRCmvMFE, Section 3 describes the experiments performed, Section 4 discusses the results, and Section 5 provides the conclusions.

## 2. Feature Extraction with IRCmvMFE

**Figure 1:**
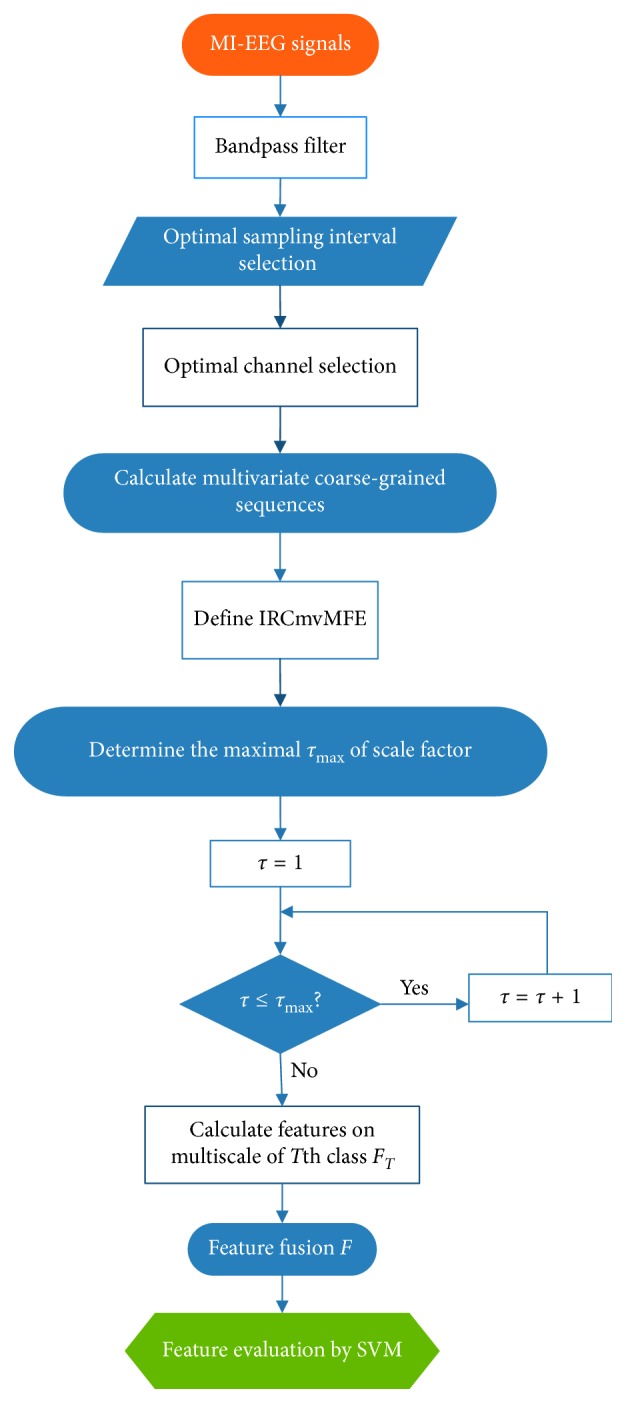
Block diagram of the proposed method.

By combining median filters and mean filters in coarse-grained processes, RCmvMFE is improved to produce IRCmvMFE, which is applied to extract features of MI-EEG. The main steps are as follows: preprocessing, optimal channel selecting, performing multivariate coarse-grained analysis of preprocessed MI-EEG data, calculating IRCmvMFE, and constructing a feature vector. The support vector machine (SVM) was used to classify the feature vector. The block diagram of the proposed method is displayed in Figure 1.

### 2.1. Preprocessing MI-EEG Signals

For two-class motor imagery tasks, assume that *X*_*T*,*C*_*i*__^0^=[*X*_*T*,*C*_*i*__^0^(1), *X*_*T*,*C*_*i*__^0^(2),…,*X*_*T*,*C*_*i*__^0^(*e*)]^*T*^ represents the *i*th channel MI-EEG sequence of the *T*th task, where *T* ∈ {1,2}, *i*=1,2,…, *p*; *e* and *p* represent the sample points and the number of total channels, respectively. *X*_*T*,*C*_*i*__^0^ is bandpass filtered to the frequency band associated with the tasks and is expressed as *X*_*T*,*C*_*i*__^1^=[*X*_*T*,*C*_*i*__^1^(1), *X*_*T*,*C*_*i*__^1^(2),…,*X*_*T*,*C*_*i*__^1^(*e*)]^*T*^. The motor imagery time period [*a*, *b*] is taken as the optimal sampling interval, and MI-EEG signals in the segment are summarized as *X*_*T*,*C*_*i*__^2^=[*X*_*T*,*C*_*i*__^2^(1),…,*X*_*T*,*C*_*i*__^2^(*N*)]^*T*^, where *N*=*b* − *a*+1 represents the sampled MI-EEG points within the optimal sampling interval.

### 2.2. Channel Selection

When the brain is engaged in motor imagery, only parts of channels are activated in the form of the power spectrum. Extracting the features of all channels not only increases the computational complexity but also increases the feature information redundancy and reduces the classification accuracy [35]. Therefore, the choice of optimal channels is important. In this paper, the Fisher score of the average power spectrum of *X*_*T*,*C*_*i*__^2^ is calculated to select channels according to the following equation:

(1) $$\begin{array}{c} F\left(i\right)=\frac{{\left|P_{1}\left(i\right)-P_{2}\left(i\right)\right|}^{2}}{\mathrm{var}\left(P_{1}\left(i\right)\right)+\mathrm{var}\left(P_{2}\left(i\right)\right)}, \end{array}$$

where *P*_1_(*i*) and *P*_2_(*i*) represent the average power spectrum on the *i*th channel of class 1 and class 2 motor imagery tasks, respectively. var() is the variance and *F*(*i*) represents the *i*th channel Fisher score. The larger the *F*(*i*), the greater contribution of the *i*th channel. The signals of *p*′ channels with the top *F*(*i*) are selected for subsequent research. *X*_*T*,*C*_*i*__^2^ is rewritten as *X*_*T*,*C*_*i*__=[*X*_*T*,*C*_*i*__(1),…,*X*_*T*,*C*_*i*__(*N*)]^*T*^, where *i*=1,2,…, *p*′, in which *p*′ stands for the number of selected channels.

### 2.3. Coarse-Graining of IRCmvMFE

***Step 1*.**

In the coarse-grained process of IRCmvMFE, the median filter is first performed on *X*_*T*,*C*_*i*__. Supposing the filter size is *j*=2*k* or *j*=2*k*+1, the data in the window would be sorted in ascending order with the filter output being

(2) $$\begin{array}{c} y_{T,C_{i}}\left(s\right)=\text{med}\left(X_{T,C_{i}}\left(s\right)\right)=\left\{\begin{array}{cc} X_{T,C_{i}}\left(k+1\right), & j=2k+1,\\ \frac{1}{2}\left[X_{T,C_{i}}\left(k\right)+X_{T,C_{i}}\left(k+1\right)\right], & j=2k, \end{array}\right. \end{array}$$

where *s* ∈ {1,…, *N*} and *X*_*T*,*C*_*i*__(*k*) means the *k*th maximum value in the window.

***Step 2*.**

For the scale factor *τ*, the *k*th coarse-grained sequence on *C*_*i*_ channel of *T*th class task is

(3) $$\begin{array}{c} y_{T,C_{i}}^{k,\tau }\left(j\right)=\frac{1}{\tau }\overset{j*\tau +k-1}{\underset{s=\left(j-1\right)\tau +k}{\sum }}y_{T,C_{i}}\left(s\right),\;1\leq j\leq N^{\prime },1\leq k\leq \tau , \end{array}$$

where *N*′=int[*N*/*τ*] represents the sample points of the coarse-grained sequence. Therefore, *τ* multivariate coarse-grained sequences are obtained and described as *Y*_*T*,*C*_*i*__^1,*τ*^=[*y*_*T*,*C*_*i*__^1,*τ*^(1), *y*_*T*,*C*_*i*__^1,*τ*^(2),…, *y*_*T*,*C*_*i*__^1,*τ*^(*N*′)], *Y*_*T*,*C*_*i*__^2,*τ*^=[*y*_*T*,*C*_*i*__^2,*τ*^(1), *y*_*T*,*C*_*i*__^2,*τ*^(2),…, *y*_*T*,*C*_*i*__^2,*τ*^(*N*′)],…, *Y*_*T*,*C*_*i*__^*τ*,*τ*^=[*y*_*T*,*C*_*i*__^*τ*,*τ*^(1), *y*_*T*,*C*_*i*__^*τ*,*τ*^(2),…, *y*_*T*,*C*_*i*__^*τ*,*τ*^(*N*′)].

### 2.4. IRCmvMFE Calculation

***Step 1*.**

The multivariate coarse-grained sequence *Y*_*T*,*C*_*i*__^*k*,*τ*^ is executed for multivariate embedded reconstruction, with the multivariate composite delay vectors *Z*_*T*,*m*_^*k*,*τ*^(*i*) calculated as

(4) $$\begin{array}{c} Z_{T,m}^{k,\tau }\left(i\right)=\left[y_{T,C_{1}}^{k,\tau }\left(i\right),\ldots ,y_{T,C_{1}}^{k,\tau }\left(i+\left(m_{1}-1\right)\lambda_{1}\right),y_{T,C_{2}}^{k,\tau }\left(i\right),\ldots ,y_{T,C_{2}}^{k,\tau }\left(i+\left(m_{2}-1\right)\lambda_{2}\right),\ldots ,y_{T,C_{p^{\prime }}}^{k,\tau }\left(i\right),\ldots ,y_{T,C_{p^{\prime }}}^{k,\tau }\left(i+\left(m_{p^{\prime }}-1\right)\lambda_{p^{\prime }}\right)\right], \end{array}$$

where *i* ∈ [1, *N*′ − *n*] and *M*=[*m*_1_, *m*_2_,…, *m*_*p*′_] and *λ*=[*λ*_1_, *λ*_2_,…, *λ*_*p*′_] are the embedding dimension vector and time delay vector, respectively. Additionally, *m*=∑_*i*=1_^*p*′^ *m*_*i*_ and *n*=max{*M*} × max{*λ*}.

***Step 2*.**

The distance of any two multivariate composite delay vectors *Z*_*T*,*m*_^*k*,*τ*^(*i*) and *Z*_*T*,*m*_^*k*,*τ*^(*j*) is computed in the following equation:

(5) $$\begin{array}{c} d\left[Z_{T,m}^{k,\tau }\left(i\right),Z_{T,m}^{k,\tau }\left(j\right)\right]=d_{T,m}^{ij,k,\tau }=\max\left\{\left|y_{T,C_{g}}^{k,\tau }\left(i+l-1\right),y_{T,C_{h}}^{k,\tau }\left(j+l-1\right)\right|,l=1,2,\ldots ,m\right\}, \end{array}$$

where *i*, *j* ∈ [1, *N*′ − *n*], *i* ≠ *j*, and *g*, *h* ∈ [1, *p*′].

***Step 3*.**

Given a threshold *r*, suppose the fuzzy membership function is *μ*(*x*, *r*)=*e* ^−*d*^2^/*r*^, the similarity *D*_*T*,*m*_^*ij*,*k*,*τ*^ between *Z*_*T*,*m*_^*k*,*τ*^(*i*) and *Z*_*T*,*m*_^*k*,*τ*^(*j*) is

(6) $$\begin{array}{c} D_{T,m}^{ij,k,\tau }=\mu \left(d_{T,m}^{ij,k,\tau },r\right)=\exp\left(\frac{-{\left(d_{T,m}^{ij,k,\tau }\right)}^{2}}{r}\right). \end{array}$$

***Step 4*.**

The average membership grade *ϕ*_*T*,*m*_^*k*,*τ*^(*r*) can be obtained using the following equation:

(7) $$\begin{array}{c} \phi_{T,m}^{k,\tau }\left(r\right)=\frac{1}{N^{\prime }-1}\overset{N^{\prime }-n}{\underset{i=1}{\sum }}\frac{\sum_{j=1,j\neq i}^{N^{\prime }-1}D_{T,m}^{ij,k,\tau }}{N^{\prime }-n-1}. \end{array}$$

***Step 5*.**

Repeat the above steps, extend the dimension of the multivariate composite delay vector from *m* to *m*+1 and derive *ϕ*_*T*,*m*+1_^*k*,*τ*^. For each *Z*_*T*,*m*_^*k*,*τ*^(*i*), we get *τϕ*_*T*,*m*_^*k*,*τ*^(*r*) and *τϕ*_*T*,*m*+1_^*k*,*τ*^(*r*). The average ${\bar{\phi }}_{T,m}^{k,\tau }\left(r\right)$ and ${\bar{\phi }}_{T,m+1}^{k,\tau }\left(r\right)$ are calculated. The definition of IRCmvMFE is as follows:

(8) $$\begin{array}{c} \text{IRCmvMFE}\left(X_{T,C_{i}},M,\tau ,n,r\right)=-\ln\left[\frac{{\bar{\phi }}_{T,m+1}^{\tau }\left(r\right)}{{\bar{\phi }}_{T,m}^{\tau }\left(r\right)}\right]. \end{array}$$

The procedure for calculating IRCmvMFE is summarized in Algorithm 1.

### 2.5. Determination of a Maximum Scale Factor

As the number of scale factors increases, multivariate coarse-grained sequences become smoother. Scale factors that are too large omit useful information and reduce classification accuracy. Therefore, the impact on sequence smoothness and classification accuracy should be considered comprehensively to determine its maximum scale factor *τ*_max_.

### 2.6. Construction of a Feature Vector

For *τ* ∈ [1, *τ*_max_], IRCmvMFE at *τ* scale in the *T*th class task, i.e., IRCmv_*T*_^*τ*^, is estimated and combined to form the feature vector *F*_*T*_:

(9) $$\begin{array}{c} F_{T}=\left[{\text{IRCmv}}_{T}^{1},{\text{IRCmv}}_{T}^{2},\ldots ,{\text{IRCmv}}_{T}^{\tau_{\max}}\right]\in R^{\tau_{\max}}. \end{array}$$

The feature vectors of the two tasks are fused in parallel to obtain the feature vector of MI-EEG:

(10) $$\begin{array}{c} F=\left[\begin{array}{c} F_{1}\\ F_{2} \end{array}\right]\in R^{2\times \tau_{\max}}. \end{array}$$

## 3. Experimental Research

### 3.1. Data Description and Preprocessing

**Figure 2:**
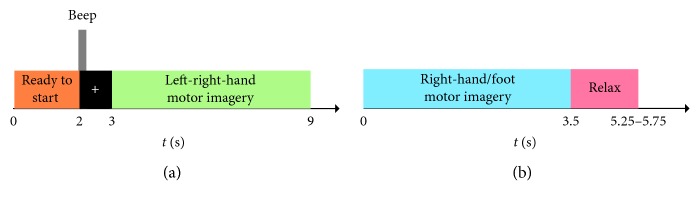
Timing scheme of MI-EEG collection. (a) Left-right-hand motor imagery task. (b) Right-hand/foot motor imagery task.

MI-EEG data were obtained from dataset III in the BCI Competition II [36] and dataset IVa in the BCI Competition III [37]. MI-EEG signals on channels C3, Cz, and C4 were recorded in dataset III of BCI Competition II, where the data were from a healthy subject who imagined left-right hand movement. Left- and right-motor imagery tasks were each performed 140 times for a total of 280 experimental trials. The signals were sampled at 128 Hz and filtered to 0.5–30 Hz. The MI-EEG collection timing scheme is shown in Figure 2(a). The subject was at rest for the first 2 s, and the corresponding motor imagery task was completed according to the screen prompts from 3 s to 9 s. To better distinguish the two-class tasks, this paper used the sampling interval [451, 900].

The dataset IVa of BCI Competition III recorded the MI-EEG signals of five healthy subjects using 118 channels during right-hand (RH) and right-foot (RF) motor imagery tasks. The original sampling rate was 1000 Hz, but we downsampled these data to 100 Hz. The subjects performed the corresponding imaginary movement according to the prompts in the first 3.5 s and then rested for a random epoch between 1.75 s and 2.25 s. The timing scheme of MI-EEG collection during the right-hand-foot motor imagery task is shown in Figure 2(b). Each subject performed 280 trials, with 140 each of the RH and RF motor imagery tasks. In this paper, MI-EEG related to mu rhythm (8–13 Hz) and beta rhythm (14–32 Hz) related to motor imagery tasks were selected, i.e., the original MI-EEG signals were preprocessed by a bandpass filter of 8–32 Hz. The data between 0.5 s and 3.5 s were used for subsequent experimental research.

### 3.2. Channel Selection

**Figure 3:**
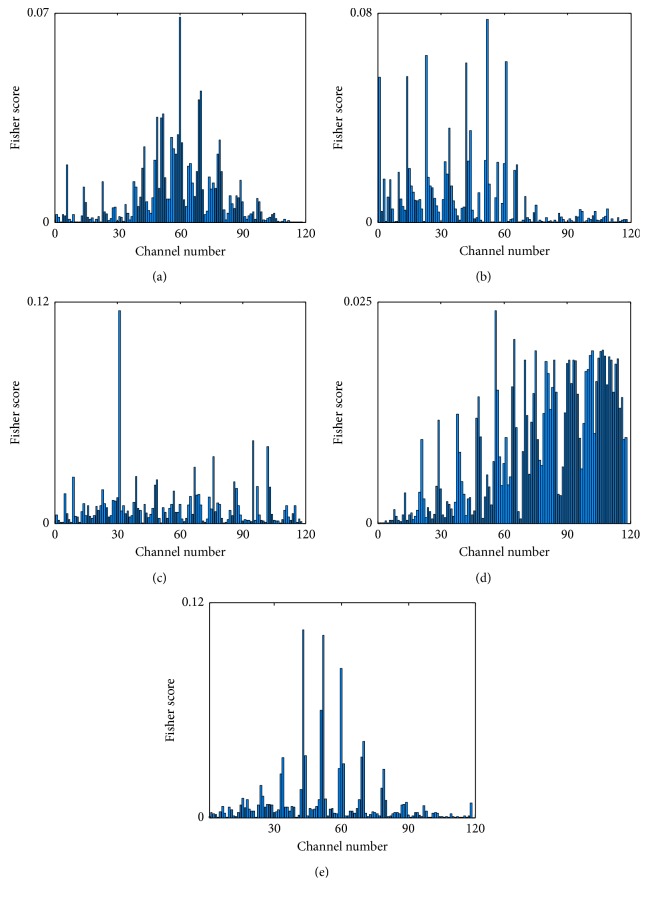
The Fisher scores of all channels with different subjects. (a) Subject “aa.” (b) Subject “al.” (c) Subject “av.” (d) Subject “aw.” (e) Subject “ay.”

Channel selection directly affects the quality of feature information and classification accuracy. It is essential to select the optimal channels before extracting MI-EEG features. There was a close relationship between the signals on channels C3, Cz, and C4 in the left-right-hand motor imagery task, so the data of these three channels were used for feature extraction. When RH and RF motor imagery tasks were conducted in dataset IVa from BCI Competition III, the Fisher Score of each channel was calculated by equation (1). The scores of different subjects are shown in Figure 3.

For each subject, the score of each channel is different and for different subjects, scores from the same channel are different. Thus, the optimal channels for each subject are different due to individual differences. The channels with the top three Fisher scores can be used as the optimal channels. The detailed information is shown in Table 1.

### 3.3. Comparison of Coarse-Grained Sequences between IRCmvMFE and RCmvMFE Methods

**Figure 4:**
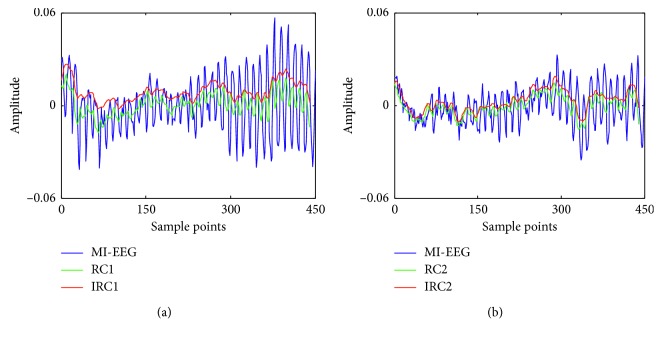
Comparison of MI-EEG and the coarse-grained sequences using RCmvMFE and IRCmvMFE during (a) left-hand motor imagery task and (b) right-hand motor imagery task.

**Figure 5:**
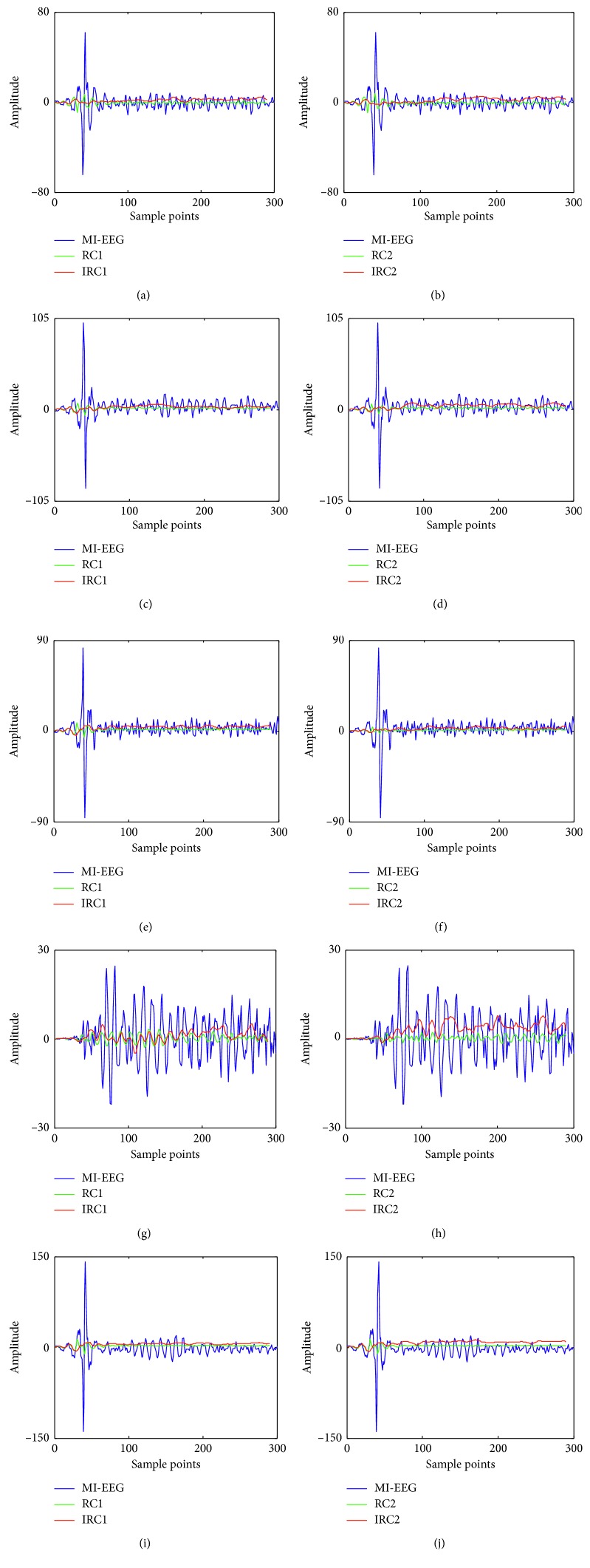
Comparison of MI-EEG signals and the coarse-grained sequences by RCmvMFE and IRCmvMFE during RH and RF motor imagery tasks. (a) Subject “aa” with RH motor imagery. (b) Subject “aa” with RF motor imagery. (c) Subject “al” with RH motor imagery. (d) Subject “al” with RF motor imagery. (e) Subject “av” with RH motor imagery. (f) Subject “av” with RF motor imagery. (g) Subject “aw” with RH motor imagery. (h) Subject “aw” with RF motor imagery. (i) Subject “ay” with RH motor imagery. (j) Subject “ay” with RF motor imagery.

To confirm the effectiveness of IRCmvMFE in extracting MI-EEG features, the coarse-grained processes of RCmvMFE and IRCmvMFE were compared. The relevant parameters were selected as follows: *m*_*k*_=2, *λ*_*k*_=1, *r*=0.2SD,  and *τ*=10, where SD represents the standard deviation of *X*_*T*,*C*_*i*__. According to Table 1, the channel *k* with the highest Fisher score of each subject was selected. The experimental process was as follows: when a motor imagery task was performed, at *τ* scale, the first *j*(0 ≤ *j* ≤ *N* − *τ*) points of *X*_*T*,*k*_ were removed in turn. The RCmvMFEs of the remaining points were calculated separately, and they were composed of time series recorded as RC_*T*,*k*,*i*_=[RC_*T*,*k*,*i*_(1), RC_*T*,*k*,*i*_(2),…, RC_*T*,*k*,*i*_(*N* − *τ*+1)], where *T* ∈ {1,2}, *i* ∈ [1, *n*_*e*_], and *n*_e_ represents the number of experiments. The RC_*T*,*k*,*i*_ of *n*_e_ experiments were superimposed and averaged to obtain the average time series RC_*T*,*k*_. The average time series of IRCmvMFE was obtained the same way as IRC_*T*,*k*_. When imaging left-right-hand motor imagery, training set data were used for analysis, i.e., *n*_e_=70. Similarly, *n*_e_ was selected as 140 when the RH and RF motor imagery were performed. The amplitude of the original MI-EEG signals and coarse-grained sequences of RCmvMFE and IRCmvMFE during left-right-hand motor imagery are displayed in Figure 4. Similarly, the experimental results from imaging right-hand-foot movement are shown in Figure 5.

It can be seen from Figure 4 that the original MI-EEG signals had larger fluctuations, which was obviously improved after the coarse-grained process of both RCmvMFE and IRCmvMFE; and the smoothness of IRCmvMFE was better than RCmvMFE. In Figure 5, there are different intensity impulse noise interferences for different subjects. The coarse-grained sequences of both RH and RF motor imagery tasks using the RCmvMFE and IRCmvMFE of each subject changed with the fluctuations of the original MI-EEG but oscillated more smoothly. For subject “aw,” the impulse noise is not obvious, and the coarse-grained sequence of IRCmvMFE had larger fluctuations than that of RCmvMFE. But the intensity of impulse noise interference is higher for other subjects. Both RC_*T*,*k*_ and IRC_*T*,*k*_ showed better smoothness, and IRC_*T*,*k*_ was superior to RC_*T*,*k*_ for rapid MI-EEG changes. The reason is that the coarse-grained process of RCmvMFE is equivalent to a mean filter, which has the effect of low-pass filtering and smoothing and can remove some random interference. However, it is helpless against impulse noise caused by sudden factors such as eye-movements, blinks, and motion. In the coarse-grained IRCmvMFE, the median filter is assigned to remove the impulse noise interference, and then the filtered signals are smoothed by a mean filter.

### 3.4. Selection of Parameters in IRCmvMFE

The parameter selection will affect the estimate of IRCmvMFE. According to equation (8), the estimation of IRCmvMFE is not only related to the preprocessed MI-EEG but also involves selecting an embedding dimension vector *M*=[*m*_1_, *m*_2_,…, *m*_*p*′_], time delay vector *λ*=[*λ*_1_, *λ*_2_,…, *λ*_*p*′_], threshold *r*, and scale factor *τ*. The selection of parameter *M* was similar to reference [32], i.e., *m*_*k*_=2. Parameter *λ* does not have any proven standards, so for simplicity, *λ*_*k*_ was selected as 1. The threshold *r* was determined as *r*=0.2SD.

**Figure 6:**
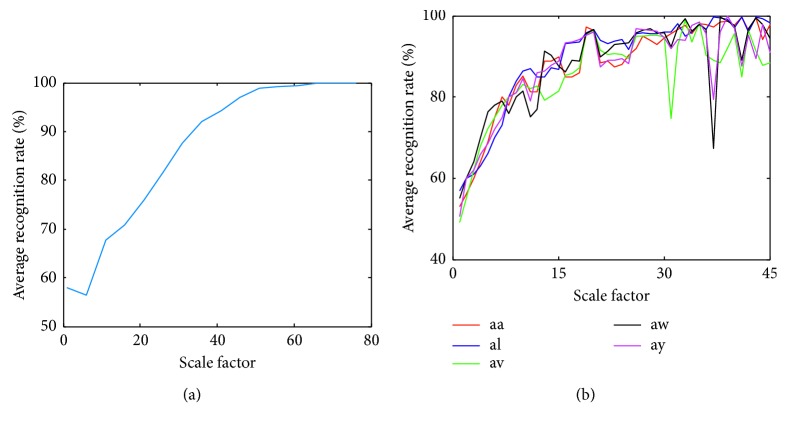
Effects of changing *τ* on the classification results during (a) left-right-hand motor imagery and (b) right-hand-foot motor imagery.

In addition, the selection of *τ* influenced the filter effect in the coarse-grained process of MI-EEG and affected the extracted features and the classification results in turn. The larger the *τ*, the larger the calculation and the better the recognition. In contrast, a smaller *τ* resulted in poor filter performance [34]. When *τ* ∈ [1,75], the IRCmvMFEs with imaging left-right-hand movements were estimated and then classified by SVM. Gaussian kernel function was employed in this paper, and SVM optimized by grid search. When *τ* ∈ [1,45], the same experiment was performed with right-hand-foot motor imagery tasks. The 10 × 10-fold cross-validation (CV) was used to eliminate the contingency in the feature extraction process of MI-EEG. The average classification accuracy of the 10 × 10-fold CV is shown in Figure 6.

In Figure 6(a), the classification results gradually increased as the scale factor *τ* increased. When *τ* was from 55 to 75, the classification accuracy tended to be stable and close to 100%, and the highest recognition was obtained at 65 scale. Therefore, the maximum *τ* about left-right-hand motor imagery was selected as 65. In Figure 6(b), with the increased *τ*, the average recognition rate of each subject first increased and then later decreased. In this paper, the *τ*_max_ values of subjects “aa”, “al”, “av”, “aw,” and “ay” during right hand-foot-motor imagery were chosen as 41, 37, 33, 38, and 39, respectively. And, *τ*_max_ is related to the mathematical model of the coarse-grained process of IRCmvMFE. There is a significant difference in *τ*_max_ during different types of two-class motor imagery tasks, while the difference between multiple subjects during the same type of tasks is not obvious.

### 3.5. Comparison of Multiple Entropy-Based Feature Extraction Methods

**Figure 7:**
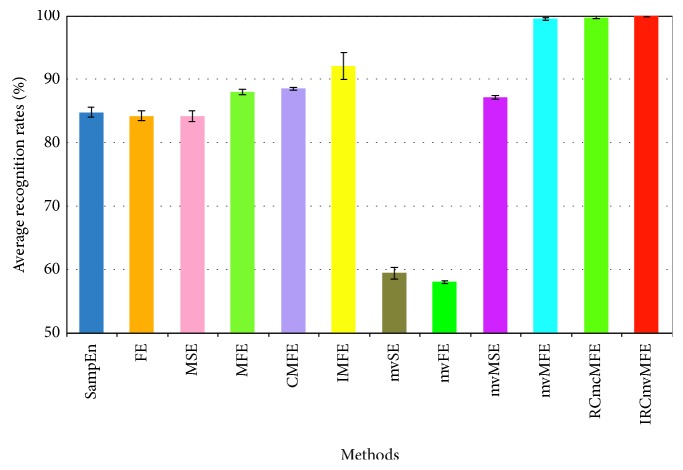
Comparison of multiple entropy-based feature extraction methods.

In this section, the comparative study of IRCmvMFE and various entropy-based feature extraction methods was conducted. To make the comparison process more objective, the same dataset was selected as reference [13, 22], i.e., dataset III from BCI Competition II, and SVM was used for classification. The classification result of IMFE was derived from [22], and the related parameters of other entropy-based methods were selected as references [13]. The average recognition results of 10 × 10-fold CV and standard deviations are displayed in Figure 7.

In Figure 7, the classification result of MFE was higher than SampEn, FE, and MSE. Because the fuzzy membership function was used to enhance the stability of MFE, richer feature information from the multiscale was collected. At the same scale, the information of multiple coarse-grained sequences was integrated by CMFE, yielding a slightly better result. Based on the parameters' independent optimization strategy, the preferred parameters were used by IMFE to extract features from the MI-EEG, and the recognition accuracy was further improved. Despite the results of mvSE, mvFE, and mvMSE being poor, mvMFE, RCmvMFE, and IRCmvMFE showed the advantages of multivariate entropy methods over traditional univariate entropies, both in terms of classification accuracy and standard deviation. This was mainly because these feature extraction methods evaluated the multivariate complexity of multichannel data and expressed the dynamic relationships and synchronizations across channels.

IRCmvMFE, RCmvMFE, and mvMFE methods displayed superiority on dataset III from BCI Competition II. To further illustrate the improvement of IRCmvMFE, a comparative study of these three methods was performed based on dataset IVa and using SVM for classification. The classification results with 10×10-fold CV are shown in Table 2. For each subject, the recognition rates obtained by using RCmvMFE to extract features of MI-EEG were higher than those of mvMFE because the multivariate feature of RCmvMFE was considered at the same scale, and the defect of unstable entropy values, i.e., coarse-grained time series shortening with scale factor increases, was overcome. Moreover, a composite filter technique was applied in the coarse-grained process of IRCmvMFE to eliminate burst-like impulse noise and the Gaussian noise of the MI-EEG, which produced better quality information. For different subjects, IRCmvMFE achieved better recognition accuracy and a smaller standard deviation than RCmvMFE, illustrating the stability and superiority of IRCmvMFE. Further, according to Figure 5, the impulse noise interference was not obvious for subject “aw,” and the recognition result by IRCmvMFE was slightly better than RCmvMFE. However, there was greater impulse noise interference for most subjects (“aa,” “al,” “av,” “ay”), after using IRCmvMFE to enhance the filter effect, the recognition results were obviously improved.

### 3.6. Statistical Analysis

In this section, statistical analysis was performed to further describe the development of IRCmvMFE. The kappa coefficient, which was designed to measure the classification precision and the comparison of performance in multiclass tasks, was made fairer. This method is a common indicator for evaluating the performance of BCI systems [38, 39]. The calculation of *κ* coefficient was expressed as

(11) $$\begin{array}{c} \kappa =\frac{p_{0}-p_{e}}{1-p_{e}}, \end{array}$$

where *p*_0_ represents the classification accuracy and *p*_*e*_ means the probability of opportunity consistency. For a two-class task, if the number of samples across classes was equal, then the value of *p*_*e*_ was 0.5. Using equation (11), the mean kappa coefficients of IRCmvMFE, RCmvMFE, and mvMFE with 10 × 10-fold CV were calculated. The results are shown in Table 3.

Comparing the mean kappa values, the results of MI-EEG feature extraction from each subject was highest when using IRCmvMFE; this result revealed that IRCmvMFE had better consistency than those of RCmvMFE and mvMFE.

### 3.7. Comparison of Multiple Traditional Feature Extraction Methods

A variety of traditional feature extraction methods [3–9] were compared with the method presented in this paper, using SVM as a classifier. In Table 4, the top classification results and average classification of 10 × 10-fold CV of referenced feature extraction methods [3–7] on BCI competition II are displayed. IRCmvMFE achieved the highest classification accuracy over the referenced methods, and its 10 × 10-fold CV results were also better; it also showed the ability of IRCmvMFE to quantify the complexity of multichannel signals and implied its superiority in extracting features from MI-EEG signals.

The CSP-based feature extraction methods have been extensively studied on BCI competition III. The experimental results of 10 × 10-fold CV with CSP, filter bank CSP (FBCSP), discriminant FBCSP (DFBCSP), sparse FBCSP (SFBCSP), and spectrally weighted CSP (SWCSP) methods were from references [8, 9]. The method presented in this paper was compared with these methods, and the recognition rates are shown in Table 5. The results of CSP-based feature extraction were lower than those of IRCmvMFE. CSP-based methods only considered the spatial characteristics of MI-EEG signals, ignoring the features in other domains. IRCmvMFE effectively extracted nonlinear dynamic features of MI-EEG, correctly analyzed multichannel signals, and had good applicability in multiple subjects.

## 4. Discussion

In this paper, IRCmvMFE was proposed as a feature extraction method for MI-EEG signals. In IRCmvMFE, a composite filter technique was applied to improve the coarse-grained process of RCmvMFE, which eliminated impulse noise interference due to random factors, produced smoother MI-EEG time series, and enhanced the filter results. The optimal channels and the optimal parameters were selected to calculate IRCmvMFE for each subject when imaging left-right-hand or right-hand-foot movement. Multiscale IRCmvMFEs were constructed as a feature vector. Entropy-based and traditionally referenced feature extraction methods were compared on two public datasets. The kappa coefficients of IRCmvMFE, RCmvMFE, and mvMFE were calculated for statistical analysis. The results implied the superiority and applicability of IRCmvMFE for the analysis of two-class motor imagery tasks. In the future, we will continue to focus on the research of multiclass motor imagery tasks.

## 5. Conclusions

A novel nonlinear dynamics method based on RCmvMFE, called IRCmvMFE, was introduced in this study. This method provides a potential tool for the nonlinear dynamic analysis of multichannel MI-EEG signals. RCmvMFE was developed using a composite filter technique in the coarse-grained process, which effectively removes impulse noise interference, better reflects the dynamic correlations both within and across channels, and is more closely matched the nonlinear and time-varying characteristics of MI-EEG and produced better features and classification accuracy. IRCmvMFE was applied to the analysis of multichannel MI-EEG signals and was compared to other commonly used feature extraction methods. IRCmvMFE yielded the highest classification results and improved stability; it also displayed the applicability of IRCmvMFE for MI-EEG feature extraction and provided a useful tool for the analysis of other complex, two-class biological signals.

## Figures and Tables

**Algorithm 1:**
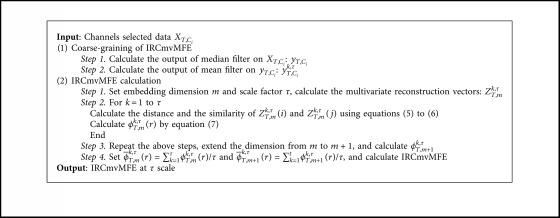
The procedure for calculating IRCmvMFE.

**Table 1:** The optimal channel combination.

Subjects	Optimal channel combination
“aa”	60, 70, 69
“al”	52, 23, 61
“av”	31, 95, 102
“aw”	56, 65, 75
“ay”	60, 52, 43

**Table 2:** Comparison of average recognition rates with 10 × 10-fold CV (%) of multivariate entropy-based feature extraction methods.

Methods	Subjects					Average results (%)
	“aa”	“al”	“av”	“aw”	“ay”	
mvMFE	75.54 ± 1.70	86.00 ± 0.98	74.46 ± 1.54	84.07 ± 1.52	79.32 ± 1.24	79.88 ± 1.40
RCmvMFE	93.82 ± 1.48	98.46 ± 0.61	95.86 ± 1.01	98.79 ± 0.72	97.04 ± 0.79	96.97 ± 0.92
IRCmvMFE	99.39 ± 0.38	99.71 ± 0.15	98.61 ± 0.62	99.61 ± 0.26	99.82 ± 0.35	99.43 ± 0.35

**Table 3:** Kappa coefficients of multivariate entropy-based methods.

Methods	Subjects					Mean
	“aa”	“al”	“av”	“aw”	“ay”	
mvMFE	0.5108	0.7200	0.4928	0.6814	0.5864	0.5983
RCmvMFE	0.8764	0.9692	0.9172	0.9794	0.9408	0.9366
IRCmvMFE	0.9928	0.9942	0.9722	0.9942	0.9986	0.9904

**Table 4:** Comparison of multiple traditional feature extraction methods on BCI competition II.

Reference number	Methods	Top classification rates (%)	Average classification rates (%)
[3]	WT	83.57	—
[3]	HHT	87.86	—
[4]	DWT	96.06	—
[5]	DTCWT	91.07	—
[6]	EMD	99.48	—
[7]	CSP	82.86	—
This paper	IRCmvMFE	100	99.86

Note: “—” represents that average recognition rate of 10 × 10-fold CV is not given in the reference.

**Table 5:** Comparison with multiple CSP-based feature extraction methods in BCI competition III.

Reference number	Methods	Subjects					Average results (%)
		“aa”	“al”	“av”	“aw”	“ay”	
[8]	CSP	79.89	97.89	70.39	92.14	92.14	86.67
[8]	FBCSP	90.39	97.82	72.54	97.21	94.54	90.50
[8]	DFBCSP	90.32	98.46	75.14	97.82	95.29	91.40
[8]	SFBCSP	91.54	98.57	77.43	97.03	94.69	92.05
[9]	SWCSP	94.2	99.2	78	97.7	95.6	93.0
This paper	IRCmvMFE	99.39	99.71	98.61	99.61	99.82	99.43

## Data Availability

Two previously reported datasets were used to support this study and are available at http://bbci.de/competition/ii/ and http://www.bbci.de/competition/iii. These datasets are cited at relevant places within the text as references [36, 37].
